# Medical competence, anatomy and the polity in seventeenth-century Rome

**DOI:** 10.1111/j.1477-4658.2007.00462.x

**Published:** 2007-09

**Authors:** Silvia De Renzi

**Affiliations:** The Open University

**Keywords:** Counter Reformation politics, early modern medicine, early modern Rome, history of anatomy, physicians’ careers

## Abstract

At the centre of this article are two physicians active in Rome between 1600 and 1630 who combined medical practice with broader involvement in the dynamic cultural, economic and political scene of the centre of the Catholic world. The city's distinctive and very influential social landscape magnified issues of career-building and allows us to recapture physicians’ different strategies of self-fashioning at a time of major social and religious reorganization. At one level, reconstructing Johannes Faber and Giulio Mancini's medical education, arrival in Rome and overlapping but different career trajectories contributes to research on physicians’ identity in early modern Italian states. Most remarkable are their access to different segments of Roman society, including a dynamic art market, and their diplomatic and political role, claimed as well as real. But following these physicians from hospitals to courts, including that of the Pope, and from tribunals to the university and analysing the wide range of their writing – from medico-legal *consilia* to political essays and reports of anatomical investigations – also enriches our view of medical practice, which included, but went beyond, the bedside. Furthermore, their activities demand that we reassess the complex place of anatomical investigations in a courtly society, and start recovering the fundamental role played by hospitals – those quintessential Catholic institutions – as sites of routine dissections for both medical teaching and research. (pp. 551–567)

In April 1624, while walking in the Vatican gardens, Pope Urban VIII was entertained by a small group discussing the latest startling natural phenomenon, a two-headed calf. With the pontiff were his secretary, his theologian and two physicians, Johannes Faber and Giulio Mancini. A few days before, Faber had received the calf from the Cardinal Nephew and, having dissected it in front of students at his home, now presented his drawings to the pope.^1^ Five months later, by papal order, Mancini called Faber to attend the autopsy of Marco Antonio De Dominis. One of the tragic figures of the Counter-Reformation, De Dominis had died in jail after controversially recanting his heretical views. Rumours of poisoning soon circulated and an autopsy was ordered, but the doctors judged it a natural death.^2^

These episodes tend to appear in unrelated historical accounts. De Dominis’ demise, including the subsequent burning of his corpse, has been explored by religious historians discussing ill-fated instances of reform in the Catholic Church.^3^ Historian of science Paula Findlen has analysed the conversation on the two-headed calf as an example of how, by becoming courtly displays, natural investigations gained unprecedented importance in the early modern period.^4^ In neither account has the presence of physicians been remarked. Much more generally, physicians are conspicuous by their absence from the history of early modern Rome, although a reasonable estimate put their number in 1656 as high as 140 out of roughly 120,000 inhabitants.^5^ Medicine's concern with the body made it one of the disciplines most directly intersecting with the cultural and religious project of the Counter-Reformation, which was centred on Rome, but the Eternal City has for long been neglected also by specialist historians of medicine. This is now changing; in addition to more specific studies,^6^ two preliminary attempts at assessing physicians in early modern Rome are now available. On the one hand, focusing on papal physicians, Richard Palmer concluded that they tended to be socially and intellectually weak. Building on this and also contrasting Rome with Padua, a leading centre in Renaissance medicine, Daniel Brownstein argued that medical practice rather than teaching attracted physicians to wealthy and cosmopolitan Rome, but that this prevented them from pursuing research-oriented activities, such as anatomy. On the other hand, Nancy Siraisi has engaged head-on with the erudite dimension of the works of late Renaissance Roman physicians, and shown how this interplayed with their wider activities.^7^ These are suggestive interpretations, but, in different ways, they look at physicians in isolation.

By reconstructing the careers of Mancini and Faber, I make physicians’ multifaceted interactions with the society of Counter-Reformation Rome the focus of my attention. Neither represents the rank and file of Roman doctors. The former became papal physician, though he is better known to art historians for a work on painting; the latter was a member of the Academy of the Lincei, Galileo's supporters in Rome, and he is prominent in studies on the astronomer.^8^ As a consequence, their medical activities have been overlooked, but precisely because in different ways they were well-connected actors in the social and cultural milieux of the city, they can offer valuable insights into how physicians built their identity and perceived competence and success at the heart of the Catholic world.

In my investigations I draw on burgeoning research on early modern physicians, spanning from quantitative investigations to in-depth case studies, and from exploration of the institutions which shaped their social identity – colleges and universities – to analysis of physicians’ contribution to broader intellectual trends, including the rise of empirical knowledge and the fortunes of antiquarianism.^9^ There is general consensus that between the late Middle Ages and the mid-seventeenth century, Italian physicians provided an influential model across Europe. However, the way they built their careers and fashioned themselves changed as the social and cultural context of the Italian states was transformed over the central decades of the sixteenth century, including the rise of ecclesiastical as opposed to lay careers, the centripetal forces of courts, and new patterns of consumption and devotion. To make sense of such changes we have to look more broadly than has been usually the case at the arenas where physicians moved, the skills they mobilized and the range of practices they were involved in.

Mancini and Faber were both foreigners and arrived in Rome in the last decade of the sixteenth century. In the first section, I reconstruct how medical education equipped them to start in the profession, including their expectations in choosing the Eternal City. In the second and third sections I follow their trajectories and this means to enter institutions as different as noble households, tribunals, hospitals, the papal court, national churches, and academies. The blossoming art production of Rome, including its impact on consumption patterns, and the political network that linked the Eternal City to the world are also important to making sense of their identity. While cultivating the intellectual and professional skills they had acquired at university, Faber and Mancini also gained others; both became art connoisseurs and, though in different ways, engaged with politics. Faber, who had a taste for antiquarianism, specialized in *materia medica* and took up anatomical investigations in earnest; but he also acted throughout his life as a political broker between Bavaria and Rome. Mancini, a shrewd economic operator, practised the art of writing advice, including on how to make a career at court and on gentlemen's education; while engaging in the centuries-old but still topical debate on the status of medicine, he forcefully argued for its pre-eminence and political role.

Taking into account all the arenas in which these physicians were active also allows us to look afresh at one of the most intensively studied aspects of early modern medicine, anatomical investigations. At the heart of those two meetings in 1624 was the act of dissecting bodies, but the first was an anatomical demonstration of a monstrous animal, the second an autopsy. While anatomical investigations are a time-honoured topic of research, historians have only recently started to appreciate the role of post-mortems. Built on different routines, they provided different knowledge; in Rome they both were widespread, but although they could intersect, they seem to have contributed differently to a physician's profile.^10^ Faber and Mancini were active practitioners, too. In addition to recovering the social and political significance of their relations with patients, I also argue for a broader definition of practice, which went beyond the bedside and included writing *consilia* as an expert witness.

Early modern Roman physicians have rarely been explored and often presented as uninspiring specimens of a discipline that elsewhere was undergoing fundamental changes. Yet, taking seriously how physicians interacted with Rome's distinctive culture, society and politics might expand our view of how medical competence was defined, here and elsewhere.

## ‘THE POPE IS VERY WELL DISPOSED TOWARDS PHYSICIANS’

Like no other city, early modern Rome had multiple functions; it was the capital of a powerful state, the hub for daring and lucrative business ventures, the site of numerous magnificent courts, and the headquarters of the Catholic drive to regain religious and intellectual control in Europe. Patronage relationships dominated the social landscape, but contemporaries regarded Rome as a place where fortunes could be made much more easily than anywhere else, especially thanks to the unique political arrangement of the papacy. Here a new patronage system was established every time a pope was elected. This dynamism was social as well as geographical.^11^ At the numerous courts of cardinals and noble families, people of the middle rank could climb the social ladder, and foreigners could relatively simply become Roman citizens.^12^ Physicians, like everyone else, took advantage. From within the church state, from other Italian states and from across the Alps, they flocked to Rome. Convents, monasteries, and large aristocratic households all demanded attending physicians, and especially in the bigger hospitals – financial hubs as well as places for assistance and medical care – physicians could combine a salary with useful networking.^13^

Hospitals loom large at the outset of both Mancini's and Faber's professional lives. Born into the well-connected family of a Siena physician, Mancini was appointed at the Santo Spirito in October 1592, five years after obtaining his degree.^14^ At the time he had a *condotta* in Viterbo, but did not like the job. During the last year of his medical education in Padua, he had been approached with an offer to become assistant to the physician of the King of Poland. Nothing came of it, but this stirred his ambition and Rome quickly appeared as an appropriate alternative, especially since the family had connections there.^15^ The job at the hospital seemed a wonderful opportunity, though Mancini was required not to practise privately and had to live in the hospital, rather disappointing conditions for a young and confident physician.^16^ Within a few months he was complaining that hospital life was bad for his health, but to leave might annoy the pope – Clement VIII – who was ‘very well disposed towards physicians’.^17^ Only in 1623, when he was appointed physician of Pope Urban VIII, does his name disappear from the hospital's payroll.^18^

At the University of Padua, renowned in Europe for the emphasis on practical teaching and its professors’ scholarship, Mancini had enjoyed the full medical curriculum.^19^ In addition to attending the lectures, he followed the learned Girolamo Mercuriale in his daily practice and became a favourite student, probably due to the remarkable breadth of his interests; for example, following the example of wealthier friends in Siena, he had developed a passion for painting, though, like them, he did not paint.^20^ Padua is also commonly associated with thriving anatomical teaching, but like his German fellow students, Mancini was disappointed by the unpredictable schedule of anatomical demonstrations. Only towards the end of his stay could he report an ‘anatomia perfettissima’, very likely performed by the renowned but erratic Fabrici.^21^ On his return to Siena in 1586 he was appointed to the chair of anatomy and surgery; the intriguing reference in a letter from Mercuriale to ‘models of the eye’ which Mancini had prepared for teaching is evidence of resourceful commitment.^22^

Padua had transformed a well-educated young man with a passion for painting into an ambitious physician with a clear view of how his profession could win him money and status. Thanks to his remarkable networking skills, he could now count on the recommendations of illustrious senior colleagues and leading *virtuosi*. However, Padua had also taught him that the status of medicine was controversial. Physicians had to respond to natural philosophers’ challenge that medicine was epistemologically weak,^23^ and Mancini would draw on these discussions when in Rome the attack came not from philosophers, but jurists. Reluctantly back in Siena, Mancini taught and practised for almost five years. Then, after the brief spell in Viterbo, he was off to the Santo Spirito.

Like other Roman hospitals, the Santo Spirito trained would-be surgeons and young physicians, and it was as an assistant physician that Faber walked its wards around 1600. Born to Protestant parents in Bamberg (Bavaria), he had been raised as a Catholic.^24^ He received a medical degree at the University of Würzburg, a city still split into two religious camps, but which the fervently Catholic Prince-Bishop Julius Echter von Mespelbrunn was trying to bring to uniformity, including by re-establishing a university. The 1587 statutes of the medical faculty testify to an effort to provide up-to-date teaching. Students often chose anatomical topics for their dissertations, while practical training included attendance at senior physicians’ consultations, study of plants, minerals and animals, and visits to apothecaries’ shops.^25^

Students in Würzburg were probably encouraged to complete their medical training abroad. A year after his degree, Faber crossed the Alps, but it was Rome, not Padua, that attracted him. Mancini had been fascinated by the job opportunity offered by the Eternal City; for the young German coming from a divided land, Rome had a clearer political and religious meaning as the centre of the Catholic world. It was common for converts to become zealous fighters for their new religion, and soon Faber's religious and political commitment became apparent. His mentor was Kaspar Schoppe, another Bavarian convert and controversial philologist; at the beginning it was erudition, including *materia medica*, that opened the doors of intellectual circles to Faber. Schoppe involved him in two typical humanist enterprises, a commentary on the portraits of illustrious men collected by the erudite Fulvio Orsini and an attack on the poor botanical knowledge of the erudite Joseph Scaliger. Just two years after Faber's arrival, he was appointed keeper of the Vatican Gardens and lecturer in *materia medica* at the university, jobs for which strong papal support was essential.

National communities helped foreigners find their way in Rome. It comes as no surprise then to find Faber as an active member of the German church, though he went beyond the usual call of duty. Soon after his arrival, he started to act as the representative in Rome of powerful German patrons and obtained on their behalf licences to read forbidden books and dispensations to marry. He also cared for weaker members of the German community in Rome and throughout his life remained pivotal in connecting Bavaria, a major but politically troubled Catholic ally, and the pope.^26^ Contemporary Protestant accounts describe him as one of the spies who, under the pretence of erudite companionship, would try to convert Protestant travellers. This may have been propaganda, but in a letter of 1626 asking for a pension, Faber did boast of his ‘reconciling the German Protestant Princes and keeping them in devotion towards this Holy See’.^27^ Political wheeling and dealing was his bread and butter.

Unlike Mancini, Faber was fascinated by activities in the hospital, especially post-mortems. Outside, they were both involved in the growing art market. Complementing learned physicians’ traditional philological and antiquarian pursuits with an interest in works of art, they were following the latest fashion in this major centre of art production. Although collecting paintings was becoming affordable by people across a wide social spectrum, it still remained one of the best ways to enter more exclusive circles. For Mancini, who traded in art with impressive determination, the search for patients coincided with his search for paintings. For example, he regularly attended the household of Cardinal Francesco Del Monte, one of the most active art patrons, who would introduce him to painters and collectors, soon to become his patients.^28^ Medicine, art – he sold his collection in 1620 – and shrewd investments, made Mancini a very wealthy man. Though on a completely different scale, Faber also collected paintings and, at ease in the community of German and Dutch painters, where he found patients, he also served as an art agent for his German patrons.^29^

As attending physicians Mancini and Faber competed, but the bedside was just one aspect of their medical expertise, which they came to define in different terms. In his account of the double-headed calf, Faber praised Mancini as a remarkable anatomist (*anatomicus insignis*).^30^ But it is not clear whether he referred to anything more specific than his Paduan training, for in Rome Mancini's interest in anatomy *per se* seems to have waned; none of his extant manuscripts is on this topic. Other aspects of the profession seemed more suited to promoting the discipline and raising his own status.

## MEDICINE AND POLITICS

As his correspondence shows, Mancini was mingling with, and attending, powerful clients, including some of the most influential cardinals. The enviable reputation he acquired was based on what many perceived as extraordinary semiotic skills, though his briskness at the bedside was also commented upon; and he rebutted firmly accusations that he was less than assiduous at the hospital.^31^ At the end of his life he was extremely well off, but before becoming papal physician he was happy to combine various sources of income; in 1616 he was working as physician of one of the jails for which he received a meagre 9 scudi every three months.^32^ His connections with the world of the law went beyond this job, and his manuscripts include reports written in his capacity as expert witness to the numerous tribunals of the city. For example, in 1609 he wrote a lengthy report as one of many physicians giving evidence in an alleged poisoning, a *cause célèbre* of the time.^33^ In 1612, he was asked to assess if a woman's miscarriage could have caused her death;^34^ in 1615 he was again involved in a poisoning case and a year later gave his opinion in a dispute over the effects of the construction of a building on air quality at the nearby Jesuit College.^35^ His advice was also sought in a case of alleged witchcraft,^36^ and he intervened – it is unclear in what capacity – in the discussions over Filippo Neri's canonization, challenging the official account of his palpitations.^37^ In these reports, doctrine and observation could be combined in various degrees; assessing the evidence of autopsies was an important skill, but it did not necessarily mean attending them.^38^ Early modern physicians’ interactions with the law has recently been studied in relation to their task as judges in cases of alleged malpractice. Giving testimony as expert witnesses in an increasingly wide range of legal cases, civil as well as criminal, was probably a more frequent routine.^39^

More generally, drawing on, and expanding on, the role of adviser that physicians had always enjoyed, in the seventeenth century medicine intersected with politics in at least two areas.^40^ A significant number of those writing on ‘reason of state’, one of the most controversial notions of contemporary political thought, were physicians, and this gives a new meaning to the old medical metaphors frequently occurring in such literature.^41^ But physicians were not only writing about the political body; they were also custodians of the prince's. Privy to the circumstances of his health, their knowledge and practice had dramatic political consequences. The exceptional position of the pope's or the prince's physician was recognized even by jurists who had for centuries attacked medicine in the so-called ‘dispute of the arts’.^42^ Here the status of the legal and medical professions was assessed with regard to their subject and the good that each could deliver to the community.

In Rome the traditional competition had acquired a specific social meaning: literary skills, which had determined the success of previous generations, were being replaced by professional competence, but legal expertise was much the most in demand and a degree in law the best key to the hundreds of offices in the papal bureaucracy and noble households. Physicians were feeling the heat. Giving his view in a conflict over precedence – one of many that characterized Roman urban life – in or before 1614, Mancini wrote a passionate essay on the status of medicine and its place in the life of a community.^43^

While rehearsing some of the better-known arguments of the long-standing dispute, Mancini added new reflections on physicians’ relations with political power. Despite having the same subject – the human being – medicine and the law are built in very different ways, he argued. Medical knowledge is based on natural philosophy and through the use of logic it gains certainty; by contrast, jurists lack both and rather possess a *cognitio historica*, knowledge of the laws that have accumulated, often disorderly, over the centuries. Medicine and the law also share a similar subdivision, including *iudiciaria* (that which relates to judging cases in courts), *consultativa*(that which relates to giving advice in individual cases) and *catthedrale* (that which relates to teaching). But revealingly focusing on *iudiciaria*, Mancini claimed that a physician's task is far more important than a jurist's on account of the quantity of people affected by the matters on which he exercises his judgement. While assessing the spread of plague, the virginity of a woman, or the madness of a defendant, a physician is dealing with issues to which everyone, including the prince, is subject; by contrast, laws, which are the dominion of jurists, do not apply universally as the prince is above them.

Yet, Mancini could not but accept that the noblest task in society is to give the law, which, following Aristotle's *Politics*, he regarded as the product of wisdom and prudence. However, contrary to what could be assumed, continued Mancini, law-giving is best achieved not by jurists, about whose education and achievements he was bitterly sarcastic, but by physicians. An imaginary example, but one that could actually occur, helped bring home the pre-eminence of physicians. With an implicit reference to the utopian political literature popular at the time, Mancini asked his reader to imagine a people who has landed on an unknown territory and should start living as a community. If both a jurist and a physician are with them, it is easy to see that the latter not the former would be of greater help in their task. Not only would his knowledge of the first principles such as how man's will and intellect interact be extremely useful. His competence as to the geographical and material circumstances of the country and the people, including air, water, winds and astrological conditions, would also be valuable with regard to how and where to build, as well as to establishing laws. Laws should always depend on consideration of the specific place and time of a country, which include both its natural settings and its astrological and even occult circumstances. These are known to a physician and not to a jurist. ‘The physician with his intelligence and prudence will be able to establish and make the law… therefore he will get close to the law-giving prince’.^44^

Mancini is no forerunner of Johann Peter Frank, the eighteenth-century advocate of medical police. Mancini's language was shaped by Aristotelian politics and ethics, read through the lens of contemporary political literature.^45^ His prince was not an enlightened ruler, but the prince above the law of theories on ‘reason of state’; his aim was to argue for physicians’ higher rank as their competence could actually prove vital to law-giving, the fundamental act of the prince. Medical knowledge meant a combination of Aristotelian natural and moral philosophy with the Hippocratic understanding that the environment has a major impact on individuals and their communities. The materialist approach to human nature, which had traditionally characterized medicine, could be valuable in political action.

Evoking the establishment of utopian communities should not mislead us; Mancini's intended audience was papal circles. However above the law, the prince/pope was subject to the vagaries of his body and the role of his physician was thus all the more important. In a society in which status and authority were measured in terms of proximity to the source of political power, the way to enhance a physician's rank was to become attending doctor of the prince, which Mancini accomplished in 1623 when he was appointed by Urban VIII. However, just a few years later he discovered to have competitors; since 1628, the pope developed a close relationship with the philosopher and astrologer Tommaso Campanella, and Mancini might have become uncomfortable.^46^ Meanwhile Faber's career was following a different path.

## DISSECTIONS AND PHYSICIANS’ IDENTITY

In 1611, Faber became a member of the Academy of the Lincei, whose commitment to natural investigations made it unique among contemporary academies. Different strands coexisted in the Lincei; in Faber the rhetoric and practice of observation, rooted in his early anatomical education, was combined with the innovative natural philosophy of Bernardino Telesio's followers, whom he had encountered on a trip to Naples in 1608. The constant supply of information about nature that reached Rome, the centre of missionary networks, also enriched his views. Faber's connections with Germany became instrumental in promoting Galileo across the Alps and he successfully acted on behalf of the founder of the Lincei, Prince Federico Cesi.^47^ Yet, to some of the wealthier members of the Academy, he often appeared more a client than a fellow academic.

Partly, this was the result of the multifaceted relations of the Academy with medicine. The Lincei's investigations on the variety and morphology of plants, animals and minerals included their medicinal virtues.^48^ Yet in Prince Cesi's austere view, natural investigations conflicted with courtly life as much as with the professions. Medicine and the law were the obvious choice of those more interested in status and money than scholarship, and physicians hunting for *condotte* (public positions) and private clients were as removed from the ideal Linceo as courtiers entertaining their patrons.^49^ The irony was that, precisely because Faber was involved in the profession, he had much easier access to the community of those pursuing research – physicians, apothecaries and surgeons – than any other Linceo.

Faber struggled throughout his life to improve his position and earn more. The chair of *materia medica* had come with one of the lowest salaries in the university, and did not become the first step in a glittering career. Already in 1612 he was begging Paul V to give him a position either among the senior physicians of the Santo Spirito, or in the pope's household.^50^ In 1624, his hopes were raised when university chairs and salaries were to be rearranged. Faber pulled all his strings to win the patronage of the Cardinal Nephew, Francesco Barberini, but to his bitter disappointment, the outcome of months of scheming was a pitiful increase in his salary.^51^ He thought that the new chair would allow him to make his anatomical observations available for the public benefit (*benefizio pubblico*), but this did not mean much to the court.^52^

Being a German created its own problems. Faber cultivated his German identity in political as well as medical terms and his links with Bavaria included correspondence with physicians who would keep him abreast of new chemical and alchemical investigations. As a result he was, behind the scenes, the fundamental actor in a bitter controversy over the use of chemical drugs that divided the community of physicians in Rome between the 1610s and 1620s. He enthusiastically involved German physicians in the dispute, but in a vitriolic attack addressed to the by now powerful Mancini, the Roman physician Pietro Castelli accused Faber of forgetting that, although a German, it was in Rome that he was making his living.^53^ Rivalry over possession of chemical knowledge – either a German or an Italian tradition – meant that even in cosmopolitan Rome national loyalty could become an issue.

Faber was at the centre of various networks, yet at times it was hard to integrate the different components of his identity, and he remained a physician among the Lincei, a Linceo at the Sapienza, and a German living in Italy. So where could he feel more at ease? It was with physicians and surgeons working in hospitals, as well as with apothecaries, that Faber collaborated most intensely. Since his own training at the Santo Spirito, he had regarded hospitals as key sites for the production and transmission of knowledge. Although boundaries between surgeons and physicians were contentious, there is evidence that their training at hospitals could partly overlap. A certificate of attendance issued to a young Swiss doctor in 1614 shows Faber mentoring physicians while practising surgery at the hospitals of San Giovanni and Santa Maria della Consolazione, and the training also included attendance at his private practice.^54^

Since the mid sixteenth century, when Bartolomeo Eustachi taught in Rome, hospitals had been the site of anatomical investigations. The abundance of corpses attracted anatomists, though it is not clear how dissections of patients related to the yearly demonstration on the corpse of an executed criminal. By the beginning of the seventeenth century, dissections were routinely carried out for teaching and research purposes, including on the causes of death; surgeons, who were often members of hospital-based dynasties, were main actors in the practice.^55^ In 1608, the surgeon and physician Prospero Cecchini dissected an eight-month foetus at the Consolazione and, in 1611, at the S. Giovanni, a man who had died of hydrophobia; on both occasions Faber was present and took notes.^56^ Hospitals were also places for therapeutic innovations. Drugs based on minerals such as vitriol, the substance at the centre of the controversy mentioned earlier, were routinely employed; and dissections of patients to observe the side effects of powerful drugs, such as mercury, were also carried out.^57^ In 1622, Faber boasted having dissected hundreds of human corpses: exaggeration apart, it is clear that post-mortems were thriving in Rome and that Faber was an avid practitioner.^58^

**Fig. 1:**
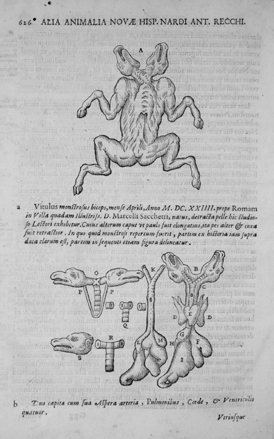
Woodcut of the double-headed calf as dissected by Johannes Faber, in his *Aliorum Novae Hispaniae Animalium Nardi Antonii Recchi Imagines et Nomina*, 1651, p. 626 (courtesy of the Whipple Library, University of Cambridge)

In his main published work, Faber recalls post-mortems he attended, but gives fuller accounts of dissections of animals, the aim of which was not to discover the causes of death or the effects of drugs, but rather to clarify the structure of the body. Faber never went to Padua, but seems fully to have embraced Fabrici's ‘Aristotle project’.^59^ To make sense of the function of organs and how physiological operations such as respiration, the development of the foetus and digestion are carried out, he dissected animals as different as calves, dogs and sea turtles and in doing so was happy to challenge the authority of leading anatomists. An outcome of this activity was his collection of skeletons, which were immortalized in the engravings of Filippo Liagno, a Neapolitan painter and friend.^60^ While this reveals an important aspect in Faber's interest in art, there is no evidence that he enjoyed a sustained co-operation with draughtsmen during his anatomical investigations. Although Prince Cesi famously employed a range of artists to illustrate his botanical research, these were not made available to Faber's enterprise and he claimed to have produced the drawings of the two-headed calf (Fig. 1).

Anatomical dissections of animals were obviously a very different activity from autopsies, and yet, as is evident now, but not at the time, they could be part of the same endeavour. During the dissection of the two-headed calf, one of the findings that most surprised Faber's students was that inflating the lungs through bellows did not cause any movement of the heart. No passage of air between the lungs and the heart could be detected. Faber commented that this happened not only in the lungs of other bigger and smaller animals, but also in those of human corpses when they were similarly inflated through bellows, ‘as will be clear to him who makes the experience’ (*experienti patebit*).^61^ The quotation suggests repeated observations undertaken with investigative as well as pedagogical intent. To do this, human cadavers were necessary and once again they were found in hospitals, where dead patients, as we have seen, were routinely opened for a range of different purposes.

However productive their relations with autopsies, anatomical investigations *à la* Fabrici interacted in a problematic way with courtly culture. Although the dissection of the monster had been carried out at the Cardinal Nephew's behest, the failure of Faber's negotiations for a better chair at the university in 1624 made painfully clear to him that showing patrons the marvellous structure of bodies was not the way to gain their support. In the biographical sketch included in the 1622 lecture I mentioned earlier, Faber downplayed the value of anatomical investigations *per se* but emphasized their value as a means to better medicine: ‘I have dissected hundreds of human corpses, not so much to learn further the very ingenious structure of members, as to explore the much hidden causes of diseases’.^62^ Whatever Faber really thought, in the 1620s to investigate the causes of death through autopsy was one of a physician's expected skills; to research the structure and function of organs was not. This was not a specifically Roman problem: the uneasy relation between anatomical research and medical practice would become the basis for clashes in the decades to come, including in Bologna.^63^

Throughout his life Faber remained a busy medical practitioner, but from his correspondence it is clear that patients were also one of the many distractions from his research. Yet it was his professional expertise at the bedside that better fit into his diplomatic and political profile. In May 1624, he successfully treated the Protestant Landgrave Georg von Hessen, who had fallen ill during a visit to Rome. There had been hopes that he would convert, and when the illustrious patient left the city in good health, Faber openly admitted to relief, since his death would have had international consequences.^64^ Protestant princes travelling to the centre of Catholicism obviously caused a lot of anxiety on both sides of the Alps and this episode gives us yet another insight into the political significance of medical practice in the suspicion-laden climate of Rome.

## CONCLUSIONS

This article has argued for an approach to the history of early modern physicians that combines a tight geographical focus with a broad scope in the analysis of where and how they built their identity. The distinctive, but very influential, social landscape of seventeenth-century Rome, which was characterized by a polycentric political configuration, a unique social dynamism, and a highly competitive environment, magnified issues of career-building and self-fashioning, making them more visible to us. Physicians abounded, but here I have focused on two. Their partly overlapping, though divergent, trajectories have made it possible to recapture the wide range of political, economic and intellectual arenas in which physicians moved. By recognizing that different ways of defining and practising medical competence were available, a richer account of physicians’ place in society has also emerged.

Although often socially insecure, physicians enjoyed a significant degree of mobility, and here I have shown how bedside practice allowed Mancini and Faber to interact with various segments of society, from hospital patients to cardinals. It also gave them access to the complex political stage of Rome. The former determinedly pursued a career as a practitioner and by reaching the top position, as papal physician, came to embody the model of the physician-adviser, whose knowledge, as he boasted, could become a useful tool for a prince. For Faber, Rome was the diplomatic and scholarly capital of the Catholic world. In this politically charged environment, everything, including a physician's success or failure at the bedside, had far-reaching consequences, as Faber, the broker between countries and faiths, soon discovered: sick, healthy and dead bodies were political as much as medical objects. Physicians had always been privy to sensitive information, but as the politics of early modern Europe became more challenging, so did their role.

Political engagement also came in the form of physicians’ expert witnessing, which complemented bedside practice, and, due to the numerous civil, criminal and religious tribunals of the city, was widespread. By taking part in one of the fundamental acts of power – administering justice – physicians could add a new component in the construction of their authority. I have shown that it was partly on the basis of this practice that Mancini reached his conclusions about the pre-eminence of medicine; and it is not by chance that the main treatise of early modern legal medicine – Paolo Zacchia's *Quaestiones Medico-legales*– was a product of seventeenth-century Rome.

As I have demonstrated, physicians could take advantage of the city's resources in another respect. They seem to have been well equipped to share in the cultural fashion of the day, from antiquarianism to the art trade, at a time when new patterns of consumption intersected with the emergence of specific expertise. The humanist skills which were traditionally part of the medical education, including philology, allowed Faber to settle in quickly and gain academic positions; on the other hand, the passion for painting Mancini had shared with the urban elite in Siena and Padua allowed him to become an astute economic agent, cleverly combining connoisseurship and professional competence. Unlike the pedantic figure of widespread caricatures, in their case the learned physician was able to share his patients’ diversity of interests.

Together, these elements made a physician's success or failure. Compared to Mancini, the talented courtier with a bold vision for medical competence, Faber's complaints about his stagnant career sound justified. However, this is where their story has also allowed me to look at early modern anatomy from a new perspective. Anatomy had been important in the education of both, but, confirming Brownstein, Mancini quickly understood that in Rome his chances of success lay in practice, not anatomical investigations. Indeed, in the complicated politics governing posts, mastering *materia medica* and the ability to display natural spectacles did not necessarily secure success; it is quite likely that Faber's dense and technical prose about respiration, foetal membranes and digestion would have bored Urban VIII, who rather enjoyed poetry and Galileo's dialogues.

Yet, I have shown that anatomy was far from being neglected in Rome. Anatomy bridged Faber's identity as a Linceo and as a teacher, and empowered him to speak with an authoritative voice and to gain recognition, even from his condescending fellow Lincei. Anatomies of animals in courtly settings have recently attracted historians’ attention, but his accounts allow us to see a busy community of surgeons and physicians dissecting cadavers in hospitals. Boasted as the triumph of a renewed Catholic devotion, well before the ‘birth of the clinic’ these were also a fundamental site for medical teaching and research, including on new drugs, and boundaries between surgeons and physicians seem to have blurred here. Through Faber, I have shown that the model of the Renaissance anatomist comprehensively investigating the animal body and its functions was still flourishing in early seventeenth-century Rome. However, the pervasiveness of hospitals meant that the focus gradually shifted from research into the normal body to investigations into the causes of death and diseases, a line of inquiry that would become very fruitful. In Rome, it also had more immediate medical, social and political applications, as the autopsy of De Dominis clearly demonstrated.

## Footnotes

1 J. Faber, ‘Aliorum Novae Hispaniae Animalium Nardi Antonii Recchi Imagines et Nomina Ioannis Fabri Lyncei Bambergensis Philosophi, Medici, publici Professoris Romani et Summo Pontifici ab herbarijs studiis Expositione’, in *Rerum Medicarum Novae Hispaniae Thesaurus* (Romae, 1651), 460–839, 599; Faber to Federico Cesi, in G. Gabrieli (ed.), *Il carteggio linceo*, (Rome, 1996), 869–70.

2 For a brief account of the autopsy, see Faber to Galilei, in G. Galilei, *Le Opere* (Florence, 1966), Vol. XIII, 207; CavazzaS‘Marcantonio De Dominis’inDizionario Biografico degli Italiani33Rome198764250.

3 D. Cantimori, *Eretici italiani del Cinquecento e altri scritti* (Turin, 1992), 473–81.

4 P. Findlen, *Possessing Nature. Museums, Collecting, and Scientific Culture in Early Modern Italy* (Berkeley, 1994), 213–14.

5 C. Cipolla, *Public Health and the Medical Profession in the Renaissance* (Cambridge, 1976), 82; the proportion of physicians in Rome was almost three times that in Amsterdam fifteen years earlier.

6 See, for example, BonellaA L‘La professione medica a Roma tra Sei e Settecento’Roma Moderna e Contemporanea61998349366; ConfortiM‘La medicina nel *Giornale de’ Letterati* di Roma (1668–1681)’Medicina nei Secoli13/12000599112361059; ‘La sanità a Roma’, special issue of *Roma Moderna e Contemporanea*, 13 (2005).

7 R. Palmer, ‘Medicine at the Papal Court in the Sixteenth Century’, in V. Nutton (ed.), *Medicine at the Courts of Europe. 1500–1837* (London, 1990), 49–78; D. A. Brownstein, *Cultures of Anatomy in Sixteenth-Century Italy*, PhD thesis, University of California Berkeley, 1996; N. G. Siraisi, ‘*Historiae*, Natural History, Roman Antiquity, and Some Roman Physicians’, in G. Pomata and N. G. Siraisi (eds.), *Historia. Empiricism and Erudition in Early Modern Europe* (Cambridge, Mass., 2005), 325–354.

8 Mancini's *Considerazioni sulla Pittura*, first published in 1956, is a valuable testimony to the unique features of collecting in early modern Rome; see L. Spezzaferro, ‘Problemi del collezionismo a Roma nel XVII secolo’, in O. Bonfait, M. Hochmann, L. Spezzaferro and B. Toscano (eds.), *Geografia del collezionismo. Italia e Francia tra il XVI e il XVIII secolo* (Rome, 2002), 1–24. On the Lincei, G. Olmi, *L’inventario del mondo. Catalogazione della natura e luoghi del sapere nella prima età moderna* (Bologna, 1992); D. Freedberg, *The Eye of the Lynx. Galileo, His Friends, and the Beginnings of Modern Natural History* (Chicago, 2002).

9 Just to consider two different countries such as Italy and Britain, see Cipolla, *Public Health and the Medical Profession*; K. Park, *Doctors and Medicine in Early Renaissance Florence* (Princeton, 1985); BirkenW‘The social problem of the English physician in the early seventeenth century’Medical History311987201216355033010.1017/s0025727300046573PMC1139712; H. J. Cook, *Trials of an Ordinary Doctor: Joannes Groenevelt in Seventeenth-Century London* (Baltimore, 1994); N. G. Siraisi, *The Clock and the Mirror: Girolamo Cardano and Renaissance Medicine* (Princeton, 1997); B. Nance, *Turquet de Mayerne as Baroque Physician: The Art of Medical Portraiture* (Amsterdam, 2001); SiraisiN G‘History, antiquarianism, and medicine: the case of Girolamo Mercuriale’Journal of the History of Ideas64200323125112852408; M. Pelling with F. White, *Medical Conflicts in Early Modern London. Patronage, Physicians, and Irregular Practitioners, 1550–1640* (Oxford, 2003); A. Pastore, *Le regole dei corpi. Medicina e disciplina nell’Italia moderna* (Bologna, 2006); on physicians and empirical knowledge, H. J. Cook, ‘Physicians and Natural History’, in N. Jardine, J. Secord and E. C. Spary (eds.), *Cultures of Natural History* (Cambridge, 1996), 91–105; Pomata and Siraisi (eds.), *Historia. Empiricism and Erudition in Early Modern Europe.*

10 ParkK‘The criminal and the saintly body: autopsy and dissection in Renaissance Italy’Renaissance Quarterly47199413311639270, and ‘The life of the corpse: division and dissection in late medieval Europe’, *Journal of the History of Medicine and Allied Sciences*, 50 (1995), 111–132 are seminal; see also her *Secrets of Women: Gender, Generation, and the Origins of Human Dissection* (New York, 2006); SiraisiN G‘Segni evidenti, teoria e testimonianza nelle narrazioni di autopsie del Rinascimento’Quaderni storici108200171974418841617.

11 R. Ago, *Carriere e clientele nella Roma barocca* (Rome, 1990); L. Nussdorfer, *Civic Politics in the Rome of Urban VIII* (Princeton, 1992); ViscegliaM A‘Burocrazia, mobilità sociale e patronage alla corte di Roma tra ’500 e ’600. Alcuni aspetti del recente dibattito storiografico e prospettive di ricerca’Roma Moderna e Contemporanea319951155; M. A. Visceglia, ‘Figure e luoghi della corte romana’, in G. Ciucci (ed.), *Roma moderna* (Rome, 2002), 39–78. On the extraordinarily varied world of artisans working for this centre of early modern consumption, see R. Ago, *Economia barocca. Mercato e istituzioni nella Roma del Seicento* (Rome, 1998).

12 MoriE‘“Tot reges in urbe Roma quot cives”. Cittadinanza e nobiltà a Roma tra Cinque e Seicento’Roma Moderna e Contemporanea41996379401.

13 S. De Renzi, ‘“A Fountain for the Thirsty” and a Bank for the Pope: Charity, Conflicts and Medical Careers at the Hospital of Santo Spirito in Seventeenth-Century Rome’, in A. Cunningham, P. O. Grell and J. Arrizabalaga (eds.), *Health Care and Poor Relief in Counter-Reformation Europe* (London, 1999), 102–131; on hospitals in Rome, among others, A. Cavaterra, ‘L’ospedalità a Roma nell’età moderna: il caso del San Giacomo (1585–1605)’, *Sanità Scienza e Storia*, (1986), 87–123; L. Cardilli (ed.), *L’antico ospedale di Santo Spirito. Dall’istituzione papale alla sanità del terzo millennio. Convegno Internazionale di Studi Roma 15–17 maggio 2001*, (Rome, 2001–2002).

14 For a well-informed biographical account of Mancini see MaccheriniM‘Caravaggio nel carteggio familiare di Giulio Mancini’Prospettiva8619977192; M. Maccherini, ‘Ritratto di Giulio Mancini’, in O. Bonfait and A. Coliva (eds.), *Bernini dai Borghese ai Barberini. La cultura a Roma intorno agli anni Venti* (Rome, 2004), 47–57; see further S. De Renzi and D. Sparti, ‘Giulio Mancini’, in *Dizionario Biografico degli Italiani* (Rome, in press). The main source for Mancini's biography is his correspondence, now held in Siena, Archivio della Società di Esecutori di Pie Disposizioni, Famiglia Mancini (hereafter ASEPD).

15 Giulio to his brother Deifebo, November 1584, ASEPD, C XIX, 166, ff. 133–134. On Rome as an alternative, see *ibid.*, f. 208.

16 Deifebo to a cousin, 21 September and 27 November 1592, ASEPD, C XIX, 166, ff. 719 and 736.

17 Deifebo to Ippolito Agostini, 29 July 1593, cited in BartaliniR‘Siena medicea: l’accademia di Ippolito Agostini’Annali della Scuola Normale Superiore di Pisa. Classe di Lettere e Filosofia25199514751530, 1522.

18 SavioP‘Ricerche sui medici e chirurghi dell’ospedale di Santo Spirito in Sassia. Sec. XVI–XVII’Archivio della Società romana di Storia Patria251971145168.

19 J. J. Bylebyl, ‘The School of Padua: Humanistic Medicine in the Sixteenth Century’, in C. Webster (ed.), *Health, Medicine and Mortality in the Sixteenth Century* (Cambridge, 1979), 335–370.

20 A nineteenth-century inventory of manuscripts very likely belonging to Mancini includes lectures of various professors in Padua: see ASEPD, C XIX, 167, ff. 160–7. For attendance at Mercuriale's practice, see Giulio to Deifebo, November 1584, ASEPD, C XIX, 166, f. 133. In 1585, Mancini supervised the publication of Mercuriale's *De decoratione*, a work on cosmetic medicine. On Mancini's early interest in painting, see Maccherini, ‘Ritratto di Giulio Mancini’.

21 Giulio to Deifebo, 2 February 1584, ASEPD, C XIX 166, f. 107. For students’ complaints about Fabrici, see A. Favaro, *Atti della nazione germanica artista nello studio di Padova* (Venice, 1911), 170.

22 Mercuriale to Mancini, 30 January 1588, ASEPD, C XIX, 166, f. 422. For the chair, see G. Cascio Pratilli, *L’università e il Principe. Gli studi di Siena e di Pisa tra Rinascimento e Controriforma* (Florence, 1975), 74.

23 H. Mikkeli, *An Aristotelian Response to Renaissance Humanism. Jacopo Zabarella on the Nature of the Arts and Sciences* (Helsinki, 1992); N. Jardine, ‘Keeping Order in the School of Padua: Jacopo Zabarella and Francesco Piccolomini on the Offices of Philosophy’, in D. Di Liscia, E. Kessler and C. Methuen (eds.), *Method and Order in Renaissance Philosophy of Nature: The Aristotelian Commentary Tradition* (Aldershot, 1997), 183–209.

24 Faber's biography is outlined in G. Belloni Speciale, ‘Giovanni Faber’, *Dizionario Biografico degli Italiani*, 43 (Rome, 1993), 686–9.

25 F. Von Wegele, *Geschichte der Universität Wirzburg* (Würzburg, 1882), Vol. 2, 191–9. On anatomical dissertations, see P. H. Bosmans, ‘Adrien Romain’, in *Biographie Nationale de Belgique* 19, (Brussels, 1902), 848–89.

26 On Faber's political activities, see De RenziS‘Courts and conversions: intellectual battles and natural knowledge in Counter-Reformation Rome’Studies in History and Philosophy of Science271996429449; I. Baldriga, *L’occhio della lince. I primi lincei tra arte, scienza e collezionismo (1603–1630)* (Rome, 2002); I. Fosi, ‘Johannes Faber: prudente mediatore o “estremo persecutore dei Protestanti”?’ in *I primi Lincei e il Sant’Uffizio: questioni di scienza e di fede. Atti del Convegno, Roma 12–13 giugno 2003* (Rome, 2005), 189–206.

27 Gabrieli (ed.), *Il carteggio linceo*, 1095.

28 Z. Wazbinski, *Il Cardinale Francesco Maria Del Monte 1549–1626* (Florence, 1994).

29 Baldriga, *L’occhio della lince.*

30 Faber, ‘Aliorum Novae Hispaniae Animalium Nardi Antonii Recchi Imagines et Nomina’, 599.

31 On all this, see the entry on Mancini in J. N. Erythreus, *Pinacotheca Altera Imaginum Illustrium, Doctrinae vel Ingenii Laude, Virorum* (Coloniae Ubiorum, 1645), 79–82, a source to be used with caution, but which archival documents have corroborated in various instances. Evidence of Mancini's interest in bedside medicine is in a collection of miscellaneous writings on medical practice, *Vectigal medicinae practicae Julii Mancini*, British Library, Sloane 3133. Mancini's semiotic skills are discussed in C. Ginzburg, *Miti, emblemi, spie. Morfologia e storia* (Turin, 1986), 172–9.

32 J. A. F. Orbaan, *Documenti sul barocco in Roma* (Rome, 1920), 317.

33 The dossier is in Biblioteca Apostolica Vaticana (hereafter BAV), Barberiniani Latini 4316, ff. 245–90.

34 BAV, Barberiniani Latini 4317, ff. 38v.–39.

35 BAV, Barberiniani Latini 4316, ff. 215–243v; the dossier on the quality of air at ff. 375–443.

36 BAV, Barberiniani Latini 4337, ff. 24–31v.

37 Archivio della Congregazione dell’Oratorio di Roma, A. III. 2. l. On medical evidence at Filippo's canonization trial, see N. G. Siraisi, ‘La comunicazione del sapere anatomico ai confini tra diritto e agiografia: due casi del secolo XVI’, in P. Galluzzi, G. Micheli and M. T. Monti (eds.), *Le forme della comunicazione scientifica* (Milan, 1998), 419–438.

38 OlsonT P‘Caravaggio's coroner: forensic medicine in Giulio Mancini's art criticism’Oxford Art Journal2820058398, but see also De RenziS‘Witnesses of the body. Medico-legal cases in seventeenth-century Rome’Studies in History and Philosophy of Science33A20022192421224068310.1016/s0039-3681(02)00005-5.

39 G. Pomata, *Contracting a Cure. Patients, Healers, and the Law in Early Modern Bologna* (Baltimore, 1998); on legal medicine, C. Crawford, ‘Legalizing Medicine: Early Modern Legal Systems and the Growth of Medico-Legal Knowledge’, in M. Clark and C. Crawford (eds.), *Legal Medicine in History* (Cambridge, 1994), 89–116; A. Pastore, *Il medico in tribunale. La perizia medica nella procedura penale di antico regime. Secoli XVI–XVII* (Bellinzona, 1998).

40 On humanist physicians as counsellors, C. Crisciani, ‘Histories, Stories, Exempla, and Anecdotes: Michele Savonarola from Latin to Vernacular’, in Pomata and Siraisi (eds.), *Historia. Empiricism and Erudition in Early Modern Europe*, 297–324; for the later period, SollJ‘Healing the body politic: French royal doctors, history and the birth of a nation 1560–1634’Renaissance Quarterly5520021259128617378097.

41 P. Burke, ‘Tacitism, Scepticism, and the Reason of State’, in J. H. Burns (ed.), *The Cambridge History of Political Thought 1450–1700* (Cambridge, 1991), 479–98.

42 G. Di Renzo Villata, ‘Il dibattito sul primato tra scienze della natura e scienze giuridiche. Giuristi e medici a confronto tra Medioevo e Rinascimento’, in *Girolamo Cardano nel suo tempo* (Pavia, 2003), 221–61, 239.

43 ‘Della precedenza del dottore in legge e medicina e a chi convenga darla’, BAV, Barberiniani Latini 4315, ff. 271–88.

44 *Ibid.*, ff. 282–282v.

45 Other political manuscripts by Mancini are discussed in Menniti IppolitoA‘“Nella Corte di Roma, o per dir meglio /nel pubblico spedale della speranza”. Note per una lettura dall’interno della curia romana seicentesca’Annali di Storia Moderna e Contemporanea4199822143.

46 He played a rather cynical role when, as astrological prognostications about Urban VIII's imminent death were cunningly used by the pope's political opponents, he contributed to the rumours by saying that the pope lacked ‘natural heat’; see L. Amabile, *Fra Tommaso Campanella ne’ castelli di Napoli, in Roma e in Parigi* (Naples, 1881), Vol. 2, 149. Mancini himself was keen on astrological medicine.

47 S. Brevaglieri, *L’Accademia dei Lincei e il libro: editoria e cultura a Roma all’inizio del Seicento*, Tesi di Dottorato, Università degli Studi di Firenze, 2005.

48 A. Clericuzio and S. De Renzi, ‘Medicine, Alchemy and Natural Philosophy in the Early Accademia dei Lincei’, in D. S. Chambers and F. Quiviger (eds.), *Italian Academies of the Sixteenth Century* (London, 1995), 175–194; Freedberg, *The Eye of the Lynx.*

49 F. Cesi, ‘Del natural desiderio di sapere et institutione de’ Lincei per adempimento di esso’, in M. L. Altieri Biagi and B. Basile (eds.), *Scienziati del Seicento* (Milan, 1980 [1616]), 39–70, 49.

50 Gabrieli (ed.), *Il carteggio linceo*, 476–7.

51 The nerve-wracking negotiations can be followed in *ibid.*, 842–955.

52 *Ibid.*, 846.

53 The main text from which to reconstruct the dispute is P. Castelli, *Epistolae medicinales* (Rome, 1626); Castelli's accusation is in the dedication to Mancini, pages not numbered. On the controversy, see TrabuccoO‘“Delle cagioni delle febbri maligne” di G. A. Borelli. Una lettura contestuale’Giornale critico della filosofia italiana792000236280; S. De Renzi, ‘Un Linceo alla Sapienza. La natura del fuoco e dei metalli in un’orazione di Johannes Faber’, in A. Battistini, G. De Angelis and G. Olmi (eds.), *Alle origini della scienza moderna: Federico Cesi e l’Accademia dei Lincei* (Bologna, in press).

54 Fondo Faber, Biblioteca dell’Accademia dei Lincei e Corsiniana, Rome, Vol. 420, ff. 471–3.

55 On surgeons, GarofaloF‘Contributo storico allo studio dell’insegnamento dell’anatomia nella Sapienza. Documenti d’archivio’Humana Studia1950125; F. Garofalo, *Memorie inedite di Andrea Belli per la storia dell’Arcispedale della Consolazione* (Rome, 1950). On anatomy in Rome, see A. Carlino, *La fabbrica del corpo. Libri e dissezione nel Rinascimento* (Turin, 1994), who suggests that the use of hospitals for ‘public demonstrations’ only started in the late seventeenth century. For a different picture, see M. Conforti and S. De Renzi, ‘Sapere anatomico negli ospedali romani: formazione dei chirurghi e pratiche sperimentali’ in A. Romano (ed.), *La culture scientifique à Rome à l’époque moderne*(Rome, forthcoming). The overlap between anatomical demonstrations and post-mortems in hospitals is discussed in M. Azzolini, ‘Leonardo da Vinci's Anatomical Studies in Milan: A Re-Examination of Sites and Sources’, in J. A. Givens, K. M. Reeds and A. Touwaide (eds.), *Visualising Medieval Medicine and Natural History, 1200–1550* (Aldershot, 2006), 147–176.

56 Faber, ‘Aliorum Novae Hispaniae Animalium Nardi Antonii Recchi Imagines et Nomina’, 614 and 494.

57 Castelli, *Epistolae medicinales*, 168–9; P. Castelli, *Chalcanthinum Dodechaporion sive duodecim dubitationes in usu olei vitrioli. Et defensio antiquorum in arsenici atque sandarachae potu ad Raymundum Mindererum* (Rome, 1619), 7.

58 J. Faber, *Oratio qua ignis & metallorum exemplo, quam parum sciamus demonstratur*, Naples, Biblioteca Nazionale, VIII. D. 13, ff. Kk–v.

59 A. Cunningham, ‘Fabricius and the “Aristotle Project” in Anatomical Teaching and Research at Padua’, in A. Wear, R. French and I. Lonie (eds.), *The Medical Renaissance of the Sixteenth Century* (Cambridge, 1985), 195–222.

60 Baldriga, *L’occhio della lince*, 52–99.

61 Faber, ‘Aliorum Novae Hispaniae Animalium Nardi Antonii Recchi Imagines et Nomina’, 601.

62 Idem, *Oratio,*ff. Kkr.–v.

63 On Bologna, see M. Cavazza, ‘The Uselessness of Anatomy: Mini and Sbaraglia versus Malpighi’, in D. Bertoloni Meli (ed.), *Marcello Malpighi Anatomist and Physician* (Florence, 1997), 129–145.

64 Gabrieli (ed.), *Il carteggio linceo*, 858, 882, 885.

